# Isolated Injury to the Motor Branch of Ulnar Nerve Following Penetrating Trauma: A Case Report

**DOI:** 10.1002/ccr3.73373

**Published:** 2026-08-18

**Authors:** Hossein Ali Abdolrazaghi, Sajede Karimi, Amirhossein Faghihi, Alireza Naghdipur

**Affiliations:** ^1^ Department of Hand Surgery Tehran University of Medical Sciences Tehran Iran; ^2^ Department of Plastic Surgery Shahid Beheshti University of Medical Sciences Tehran Iran

**Keywords:** hand injuries, microsurgery, penetrating, peripheral nerve injuries, ulnar nerve, wounds

## Abstract

Isolated injury to the deep motor branch of the ulnar nerve at Guyon's canal is an uncommon condition, particularly following penetrating trauma, and may be overlooked because sensory function is often preserved. We report the case of a 39‐year‐old right‐handed male dental assistant who presented with progressive weakness of the left hand 3 months after sustaining a puncture injury from a sharp dental instrument. Clinical examination demonstrated weakness and wasting of the intrinsic hand muscles with intact sensation. Nerve conduction studies and electromyography localized the lesion to the deep motor branch of the ulnar nerve. Surgical exploration identified a neuroma within Guyon's canal, which was excised, followed by tension‐free epineural repair under microscopy. The patient was managed with postoperative splinting and hand therapy and showed gradual functional recovery. Electromyography demonstrated evidence of reinnervation at 6 months, and first dorsal interosseous muscle strength improved from M0 preoperatively to M4 at the 18‐month follow‐up, with resolution of Froment's and Wartenberg's signs and restoration of intrinsic hand function. This case emphasizes that isolated motor branch injury should be considered in patients with persistent intrinsic hand weakness after penetrating trauma around Guyon's canal, even in the absence of sensory deficits. Careful clinical assessment, appropriate electrodiagnostic evaluation, and surgical exploration can result in favorable functional recovery.

## Key Clinical Message

Isolated injury to the deep motor branch of the ulnar nerve at the level of Guyon's Canal is exceedingly rare, particularly following penetrating trauma. As sensory function is typically preserved, diagnosis is often delayed; therefore, careful history‐taking, detailed anatomical knowledge, and early surgical exploration are essential to achieve optimal recovery.

## 1 Introduction

The ulnar nerve is one of the major peripheral nerves of the upper limb, providing motor innervation to important muscles of the forearm and hand as well as sensory supply to the skin of the fourth and fifth fingers. In the distal forearm, it gives rise to the dorsal and palmar cutaneous branches. At the wrist, the ulnar nerve enters the Guyon's canal, where it divides into two terminal branches: the superficial volar sensory branch and the deep motor branch. The deep motor branch supplies the abductor digiti minimi, the third and fourth lumbricals, the palmar and dorsal interossei, the adductor pollicis, and part of the flexor pollicis brevis [1, 2].

Several authors have classified ulnar nerve injuries at the level of Guyon's canal into three zones. Zone 1 injuries occur before bifurcation, producing both sensory and motor deficits. Zone 2 injuries involve the deep motor branch, resulting in isolated motor deficits. Zone 3 injuries involve the superficial sensory branch, producing sensory impairment without motor weakness. Injuries in Zone 2 are most frequently associated with ganglion cyst compression or fractures of the hook of the hamate [3].

Penetrating trauma leading to isolated motor ulnar nerve injury is relatively rare, accounting for approximately 2% of cases, and typically involves the digital nerves. Only a few case reports have described ulnar nerve injury at the level of Guyon's canal due to foreign body penetration [4, 5, 6, 7, 8, 9]. In contrast, compression neuropathies, particularly those caused by ganglia, are more commonly reported [9, 10, 11, 12].

Isolated injury to the deep motor branch of the ulnar nerve can result in significant functional impairment, reducing fine motor dexterity of the hand [9]. Because sensory function remains intact, diagnosis is often challenging [7]. Here, we present a rare case of isolated deep motor branch injury in Zone 2 of Guyon's canal following an occupational sharp instrument injury.

## 2 Case History

A 39‐year‐old, right‐handed Middle Eastern male dental assistant presented to our hospital with a history of progressive weakness in his left hand. The symptoms developed following an occupational injury 3 months earlier in which he was punctured by a sharp dental instrument in his left hand. At the time, he presented to a local clinic with minor bleeding. The instrument was removed, and because the laceration was small, the wound was managed conservatively with dressing.

Over the subsequent weeks, he noted progressive wasting of the ulnar aspect of the hand, especially in the last 2 weeks, and difficulty with pincer grip and finger adduction, prompting referral to our hospital for further evaluation.

**FIGURE 1:**
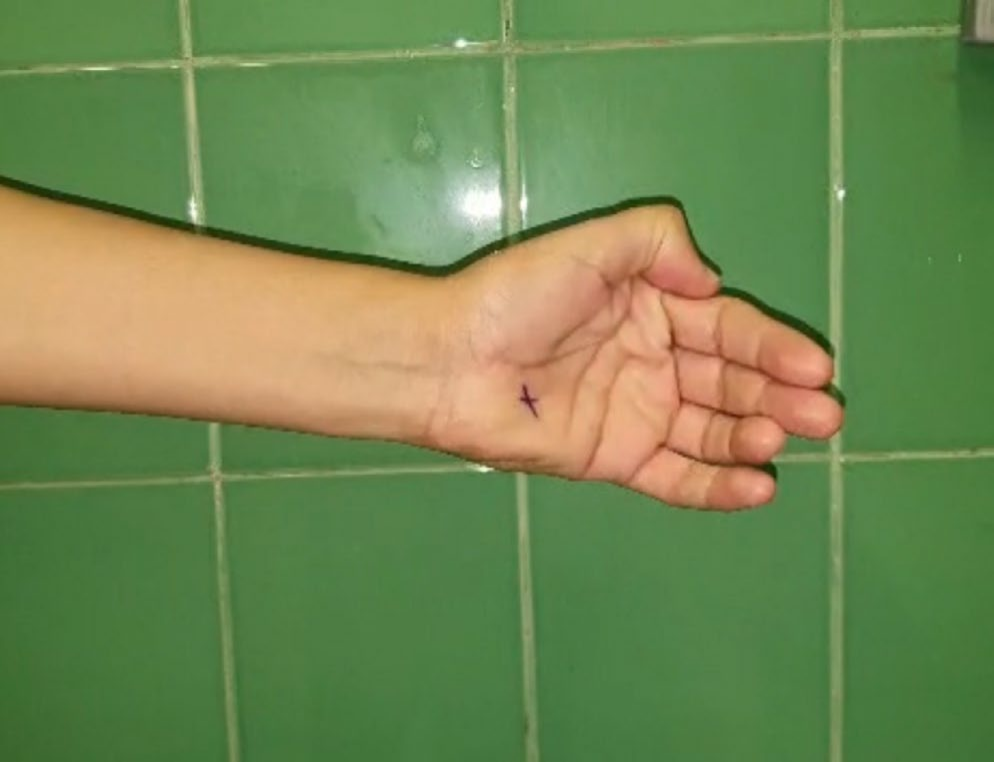
Preoperative clinical photograph of the shaved left hand demonstrating inability to make a full fist due to intrinsic muscle weakness following isolated ulnar motor branch injury. The marked area on the palm indicates the site of the initial puncture wound caused by the sharp dental instrument.

On physical examination, there was evident wasting of the hypothenar muscles, including the abductor digiti minimi, flexor digiti minimi brevis, and opponens digiti minimi. Froment's sign (the patient attempted to grasp a piece of paper between the thumb and index finger, and the examiner attempted to pull the paper away) was positive, indicating weakness of the adductor pollicis. Weakness of the dorsal and palmar interossei was demonstrated by a positive Wartenberg's sign, characterized by persistent abduction of the little finger during attempted finger adduction. In addition, there was difficulty extending the interphalangeal joints with the metacarpophalangeal joints flexed, consistent with weakness of the third and fourth lumbricals. Inrinsic hand muscle weakness caused inability to make a full fist, despite preserved finger flexion as demonstrated in Figure 1. Sensory examination, including pinprick and light touch, was intact throughout the hand, indicating preserved sensory function.

## 3 Investigations and Treatment

As the weakness developed approximately two and a half months after the trauma, nerve conduction studies were performed to exclude other causes of intrinsic hand weakness, including proximal ulnar neuropathy, cubital tunnel syndrome, and compressive lesions within Guyon's canal.

Nerve conduction studies revealed a normal ulnar sensory nerve action potential but absent compound muscle action potentials from the first dorsal interosseous. Electromyography confirmed denervation changes in the hypothenar and interosseous muscles, consistent with motor branch involvement of the ulnar nerve.

High‐resolution ultrasonography or magnetic resonance imaging was not obtained because the clinical findings, supported by electrodiagnostic studies, were sufficient to identify the site of the lesion. Since there was no evidence of a retained foreign body, ganglion cyst, or associated bony injury, additional imaging was not expected to change the treatment plan, and surgical exploration was performed to confirm the diagnosis and repair the nerve.

**FIGURE 2:**
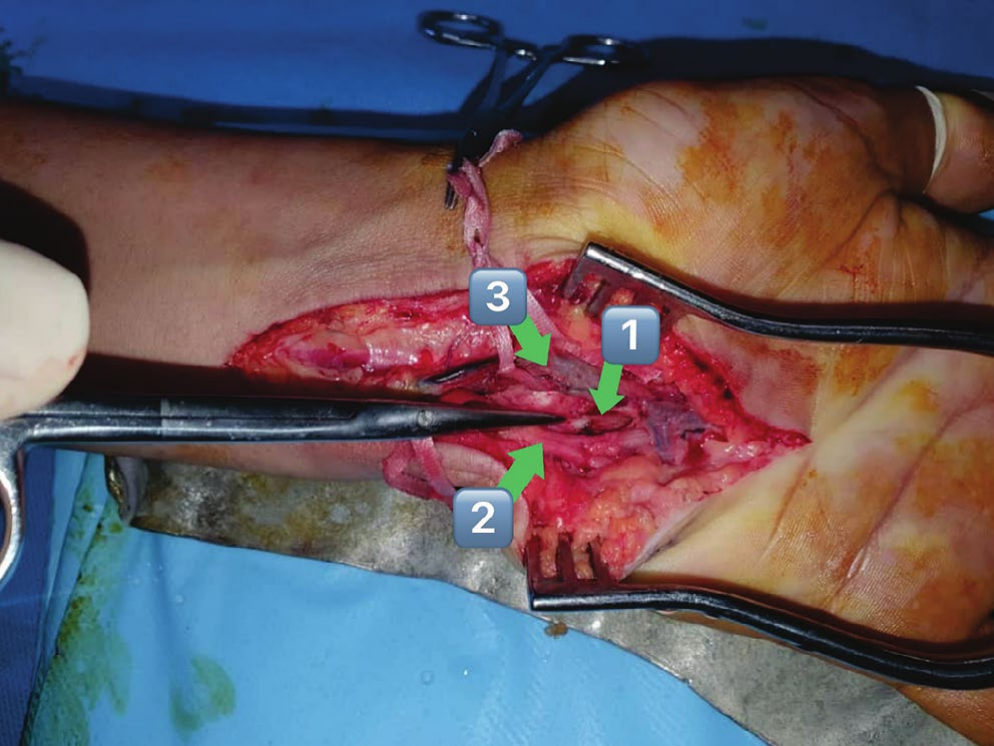
Intraoperative photograph showing a 1‐ Neuroma involving the deep branch of the ulnar nerve, 2‐ Superficial branch of ulnar nerve, 3‐ Ulnar artery, at the level of Guyon's canal, identified during surgical exploration.

Surgical exploration was performed under general anesthesia with the application of a tourniquet to minimize bleeding and optimize visualization of the surgical field. An 8‐cm longitudinal incision was made along the ulnar side of the palmar aspect of the hand. The fascia was incised radially to the flexor carpi ulnaris tendon, exposing the ulnar nerve and artery at the level of Guyon's canal. The superficial (sensory) and deep (motor) branches of the ulnar nerve were identified. Tracing the motor branch beneath the abductor digiti minimi revealed a 0.8 × 0.2 × 0.3 cm neuroma which has been shown in Figure 2. According to the principles described in Green's Operative Hand Surgery, the neuroma was resected, and the nerve was repaired with 10–0 nylon sutures using direct repair technique. Epineural repair was feasible as the resected segment allowed tension‐free approximation. The sensory branch and the ulnar artery were preserved. After completion of the repair, the tourniquet was deflated and the patient was transferred to the recovery room in stable condition.

No significant intraoperative difficulties were encountered. Careful microsurgical dissection allowed clear identification of both the superficial sensory branch and the deep motor branch while preserving the adjacent ulnar artery throughout the procedure.

## 4 Outcomes and Follow‐Up

**FIGURE 3:**
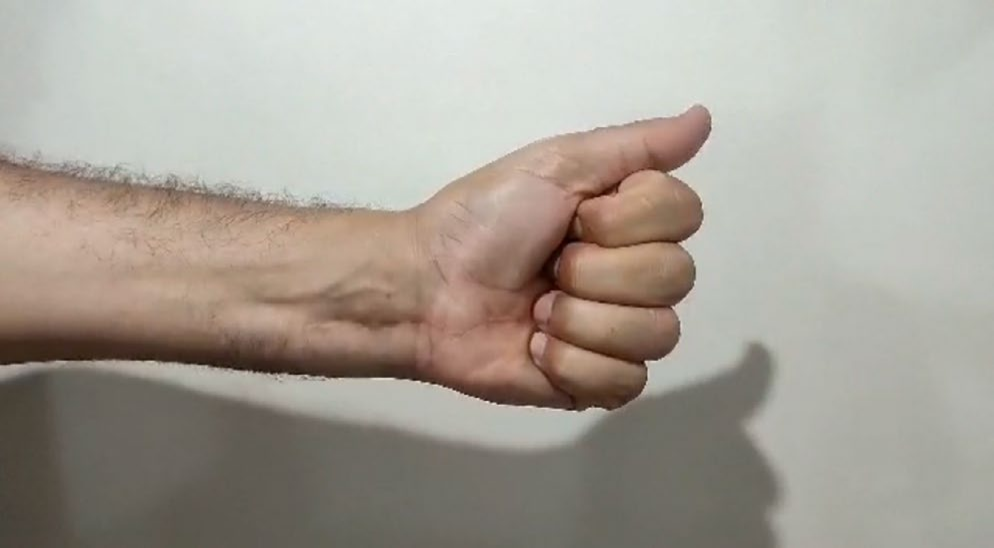
Postoperative clinical photograph demonstrating the ability to make a full fist, indicating recovery of intrinsic hand muscle function following microsurgical repair of the ulnar motor branch.

Postoperatively, the patient was advised on regular wound care, and the limb was immobilized with a splint for 21 days. Gradual mobilization of the hand and physiotherapy were initiated thereafter. Over the following months, progressive improvement in intrinsic muscle strength was observed. Electromyographic evidence of reinnervation became apparent from 6 months postoperatively. First dorsal interosseous muscle strength improved from M0 preoperatively to M1 at 6 months and gradually recovered to M4 at 18 months according to the Medical Research Council (MRC) grading system. At 18‐month follow‐up, Froment's and Wartenberg's signs were absent, with restoration of intrinsic hand muscle function and normalization of grip and pinch function on clinical examination. Also, he was able to make a complete fist, which has been shown in Figure 3.

## 5 Discussion

Ulnar nerve injuries most commonly occur at the level of the elbow, whereas lesions distal to Guyon's canal are relatively uncommon. Isolated injury to the motor branch of the ulnar nerve is exceedingly rare. Only a few reports have documented such injuries, and the majority of these are attributed to compressive etiologies, most frequently ganglion cysts or, less commonly, repetitive stress such as in cases described as “broken shovel hand” or heavy writing [9, 10, 12, 13, 14]. Penetrating trauma as a cause of isolated motor branch injury has been reported in limited studies, usually related to retained foreign bodies, iatrogenic injury during carpal tunnel release, or stab wounds [6, 7, 8, 15]. Occupational penetrating trauma caused by a slipped screw has been described in only one report [9]. The present case represents an additional example of an occupational penetrating injury, this time due to a sharp dental instrument in a dental assistant. To the best of our knowledge, this case is one of the very few reported cases of isolated ulnar motor branch injury caused by a penetrating trauma without an associated foreign body, further expanding the spectrum of mechanisms leading to this rare condition.

Diagnosis of isolated motor branch ulnar nerve injury remains challenging. Because the sensory branch is preserved and the major functions of the upper limb remain intact, such cases may go unrecognized until advanced muscle atrophy has occurred. Progressive impairment of the motor branch can ultimately lead to severe wasting of intrinsic hand muscles if diagnosis is delayed [7]. Thus, careful and detailed history‐taking is essential to ensure early recognition and better outcomes [9]. A thorough understanding of ulnar nerve anatomy and its branching pattern is also crucial for accurate diagnosis and timely management. In this case study, the patient was referred after about 3 months when marked hypothenar atrophy and significant weakness of the palmar interosseous muscles had developed.

Several studies have demonstrated that proximal ulnar nerve injuries generally carry a poorer prognosis compared with distal injuries. This difference is explained by several factors. The greater the distance between the site of injury and the target muscles, the longer the time required for axonal regeneration, thereby increasing the likelihood of irreversible muscle atrophy. In contrast, recovery after distal injuries is often more favorable because regenerating axons have a shorter distance to reach the target muscles. Earlier reinnervation helps prevent irreversible degeneration of the motor endplates and improves the likelihood of functional recovery [16, 17]. In the present case, the distal location of the injury to the Guyon's canal, combined with preservation of the sensory branch and timely surgical repair, may have contributed to the favorable functional outcome.

In this case, surgical repair was performed approximately 3 months after the initial injury. The delay occurred because the puncture wound appeared minor at presentation, sensory function remained intact, and the progressive intrinsic muscle weakness was not recognized initially. Although earlier repair is generally preferred to optimize motor recovery, previous studies have shown that satisfactory outcomes can still be achieved when repair is performed within 3–6 months, particularly for distal nerve injuries. In our patient, the excellent functional recovery was likely related to the distal location of the injury within Guyon's canal, allowing a relatively short distance for axonal regeneration and earlier reinnervation of the target muscles.

This case emphasizes the importance of early recognition of isolated ulnar motor branch injuries, particularly in occupational settings involving sharp instruments. It also highlights that penetrating injuries at the level of Guyon's canal may be easily overlooked, but distal injuries within this canal, when appropriately managed, can achieve good functional recovery despite the initial severity of motor deficits.

A limitation of this case report is that recovery was assessed using MRC grading, resolution of Froment's and Wartenberg's signs, and repeat electrodiagnostic studies, but grip/pinch dynamometry and a validated patient‐reported outcome measure (e.g., DASH/QuickDASH) were not obtained. Future cases should incorporate these standardized instruments to allow more objective, comparable assessment of functional recovery.

Future studies with larger case series and longer follow‐up are required to better define the prognostic factors affecting functional recovery after isolated deep motor branch injuries of the ulnar nerve and to compare different microsurgical repair techniques.

## 6 Conclusion

Isolated injuries of the ulnar motor branch are rare, with only a limited number of cases reported in the literature. Nevertheless, this diagnosis should be considered in patients presenting with palmar penetrating trauma, particularly when the injury occurs at the level of Guyon's canal. Early clinical suspicion and confirmation with electrophysiological evaluation can facilitate timely surgical intervention. Unlike many ulnar nerve injuries, which are often associated with poor prognosis, isolated motor branch injuries may demonstrate a more favorable outcome, with the potential for substantial functional recovery and even complete restoration of hand function.

## Author Contributions

**Hossein Ali Abdolrazaghi:** conceptualization, investigation, supervision. **Sajede Karimi:** investigation, resources, visualization, writing – original draft, writing – review and editing. **Amirhossein Faghihi:** investigation, resources, visualization, writing – original draft, writing – review and editing. **Alireza Naghdipur:** conceptualization, investigation.

## Funding

The authors have nothing to report.

## Ethics Statement

Ethical approval was not required for this single case report according to institutional policy.

## Consent

Written informed consent was obtained from the patient for publication of this case report and accompanying images.

## Data Availability

The data supporting the findings of this case report are available from the corresponding author upon reasonable request. Due to patient privacy and ethical restrictions, the data are not publicly available.

## References

[1] D. B. Polatsch , C. P. Melone, Jr. , S. Beldner , and A. Incorvaia , “Ulnar Nerve Anatomy,” Hand Clinics 23, no. 3 (2007): 283–289. 17765580 10.1016/j.hcl.2007.05.001

[2] B. Pautler , C. Marchese , M. Swancutt , and B. G. Beutel , “Anatomical Characterization and Topographic Mapping of the Distal Ulnar Nerve and Its Peripheral Branches: A Cadaveric Analysis,” Hand 21, no. 3 (2024): 402–409. 39704339 10.1177/15589447241306151 PMC11660100

[3] B. L. Maroukis , T. Ogawa , S. A. Rehim , and K. C. Chung , “Guyon Canal: The Evolution of Clinical Anatomy,” Journal of Hand Surgery 40, no. 3 (2015): 560–565. 25446410 10.1016/j.jhsa.2014.09.026 PMC4791630

[4] A. Ploumis , A. G. Christodoulou , I. Terzidis , and E. Bibasi , “Delayed Ulnar Neuropathy and Infection due to a Radiolucent Foreign Body in the Hypothenar Eminence,” Orthopedics 30, no. 2 (2007): 163–165. 17323641 10.3928/01477447-20070201-03

[5] E. V. Craig , “Delayed Laceration of Ulnar Nerve Following Hand Trauma,” Journal of the American Medical Association 253, no. 7 (1985): 1014. 3881615

[6] A. Kitay and S. S. Wolfe , “Isolated Laceration of the Deep Motor Branch of the Ulnar Nerve by a Retained Foreign Body,” American Journal of Orthopedics 41, no. 8 (2012): 371. 22900249

[7] T. Yokoi , T. Uemura , M. Okada , K. Saito , E. Onode , and H. Nakamura , “Nerve Grafting for Isolated Injury to the Intrinsic Motor Branch of the Ulnar Nerve due to a Stab Injury: A Case Report,” Journal of Hand and Microsurgery 15, no. 5 (2023): 395–398. 38152678 10.1055/s-0042-1749442 PMC10751202

[8] W. Siriwittayakorn , W. Fongsri , K. Watatham , and W. Atthakorn , “The Isolated Motor Branch of the Ulnar Nerve Injury During Open Carpal Tunnel Release,” Cureus 15, no. 8 (2023): e43601. 37719604 10.7759/cureus.43601 PMC10504059

[9] V. Khodulev , A. Klimko , O. Pereverzeva , et al., “Unusual Motor Hand Neuropathies: Causes, Diagnosis, and Evaluation of Motor Impairments,” Cureus 16, no. 8 (2024): e66381. 39246874 10.7759/cureus.66381 PMC11380553

[10] L. Asprec , M. Freedman , P. Beredjiklian , and G. Young , “Isolated Neuropathy of the Motor Branch of the Ulnar Nerve Sparing the Hypothenar Muscle and Ulnar Intrinsic Groups due to Compressive Lesion: A Case Report,” International Journal of Physiatry 5, no. 1 (2019): 17.

[11] F. Ginanneschi , G. Filippou , P. Milani , A. Biasella , and A. Rossi , “Ulnar Nerve Compression Neuropathy at Guyon's Canal Caused by Crutch Walking: Case Report With Ultrasonographic Nerve Imaging,” Archives of Physical Medicine and Rehabilitation 90, no. 3 (2009): 522–524. 19254622 10.1016/j.apmr.2008.09.568

[12] M. Saracco , R. M. Panzera , B. Merico , F. Madia , A. Pagliei , and L. Rocchi , “Isolated Compression of the Ulnar Motor Branch due to Carpal Joint Ganglia: Clinical Series, Surgical Technique and Postoperative Outcomes,” European Journal of Orthopaedic Surgery and Traumatology 31, no. 3 (2021): 579–585. 33068166 10.1007/s00590-020-02807-y PMC7981310

[13] J. D. Jennings and J. F. Jennings , “Isolated Compression of the Ulnar Nerve Motor Branch: A Case Series With 3 Unique Etiologies,” Annals of Plastic Surgery 80, no. 5 (2018): 529–532. 29489540 10.1097/SAP.0000000000001406

[14] A. Duggal , D. Anastakis , D. Salonen , and E. Becker , “Compression of the Deep Palmar Branch of the Ulnar Nerve by a Ganglion: A Case Report,” Hand 1, no. 2 (2006): 98–101. 18780033 10.1007/s11552-006-9008-0 PMC2526024

[15] R. E. Zaworski , “Undiagnosed Deep Ulnar Nerve Paralysis Resulting From Stab Wounds of the Palm,” Journal of Trauma: Injury, Infection, and Critical Care 19, no. 12 (1979): 957–960. 10.1097/00005373-197912000-00004 513176

[16] A. Semaya , “Reconstruction of High Ulnar Nerve Lesions by Distal Double Neurotization Using Motor and Sensory Branches From the Median Nerve,” Egyptian Orthopaedic Journal 50, no. 2 (2015): 122–126.

[17] A. C. Ruijs , J.‐B. Jaquet , S. Kalmijn , H. Giele , and S. E. Hovius , “Median and Ulnar Nerve Injuries: A Meta‐Analysis of Predictors of Motor and Sensory Recovery After Modern Microsurgical Nerve Repair,” Plastic and Reconstructive Surgery 116, no. 2 (2005): 484–494. 16079678 10.1097/01.prs.0000172896.86594.07

